# Air pollution and chronic bronchitis: the evidence firms up

**DOI:** 10.1136/thoraxjnl-2021-216883

**Published:** 2021-03-23

**Authors:** Frank Kelly

**Affiliations:** Environmental Research Group, Imperial College London Faculty of Medicine, London, UK

**Keywords:** COPD exacerbations

Chronic bronchitis is defined epidemiologically as cough and sputum production for ≥3 months in each of at least two consecutive years.^1^ It affects about a third of patients with chronic obstructive pulmonary disease but also occurs in individuals with normal lung function, with prevalence estimates varying widely.^2–7^ While it is not, by itself, associated with a substantially higher risk of death,^8^ it is associated with exacerbations of disease, incapacity and poor quality of life. The identification of chronic bronchitis was standardised in the first instance by the British Medical Research Council questionnaire,^9^ and subsequently, the questions were introduced into other respiratory symptom questionnaires.

Ambient air pollution is increasingly being linked to a range of human conditions, and in the case of chronic bronchitis, which affects over two million individuals in the UK,^10^ there is some evidence of an association between the incidence or prevalence of chronic bronchitis and long-term exposure to air pollution.^11–13^ Early reports, however, relate to a time when the bulk of outdoor air pollution came from coal burning when pollutant levels were considerably higher than they are today. Of interest, a real-world study by Holland and Reid showed higher rates of cough and phlegm and lower lung function in London postal workers where pollution levels were much higher than in the three county towns where comparative postal workers were recruited.^14^

In 2016, the Committee on the Medical Effects of air Pollutants identified 36 studies that examined a possible link between air pollution (primarily particulate matter (PM) pollution) and chronic bronchitis.^15^ Some of the longitudinal studies in Europe and USA that were included in this report demonstrated changes in symptoms following a reduction in the concentration of air pollution.^16–18^ However, the overall body of evidence of associations between chronic bronchitis and long-term exposure to air pollution was inconsistent, and more detailed and well-controlled epidemiological studies were recommended.^15^ Rising to this challenge, Doiron and colleagues in this issue of *Thorax* report findings from the largest analysis to date to examine cross-sectional and longitudinal associations between ambient air pollution and chronic bronchitis.^19^

In their carefully controlled study, using the Dutch Lifetimes cohort data on 132 595 (baseline) and 65 009 (second assessment) participants, they assessed possible associations of air pollution with prevalence and incidence of chronic bronchitis (winter cough and sputum almost daily for ≥3 months/year), chronic cough (winter cough almost daily for ≥3 months/year) and prevalence of cough and sputum symptoms. Logistic regression models were deployed to adjust for sex, age, educational attainment, body mass index, smoking status, pack-years smoking and environmental tobacco smoke at home. Whereas the majority of previous studies focused on exposure to particulate pollution alone, Doiron *et al* used land use regression model exposure estimates from the ELAPSE study for PM with a diameter of less than 2.5 µm, and nitrogen dioxide (NO_2_)^20^ and black carbon (BC) from the ESCAPE study.^21^

In the north of the Netherlands, NO_2_ and BC concentrations were found to be associated with increased odds of prevalent and incident chronic bronchitis. Of note, higher effect sizes were seen among women, never smokers and younger individuals supporting earlier findings for women in the European Community Respiratory Health Survey, where NO_2_ was associated with the prevalence and new onset of chronic phlegm and chronic productive cough.^22^ This finding also aligns with Gundersen *et al*, who found a large excess of cough and phlegm associated with moderate and high levels of exposure to traffic among women, which in this study was particularly marked among smokers and not found among men.^23^

Given this is the largest study to date examining the relationship between relatively low ambient air pollution exposures and chronic bronchitis, it contributes to an urgent need for research into the link between air pollution and this chronic condition. Furthermore, in relation to the impact on health, the 2005 report of the cost–benefit analyses for the Clean Air for Europe programme estimated (in monetary valuation) that chronic bronchitis was, after adult mortality, the next most important health outcome in relation to long-term exposure to air pollution.^24^ The findings reported by Doiron and colleagues set the scene for further work to estimate economic costs of this disease attributable to ambient air pollution, which, given its prevalence, are likely to be sizeable.

## References

[1] Definition and classification of chronic bronchitis for clinical and epidemiological purposes. A report to the medical Research Council by their Committee on the aetiology of chronic bronchitis. Lancet 1965;1:775–9. 4165081

[2] Cerveri I , Accordini S , Verlato G , et al . Variations in the prevalence across countries of chronic bronchitis and smoking habits in young adults. Eur Respir J 2001;18:85–92. 10.1183/09031936.01.00087101 11510810

[3] Lu M , Yao W , Zhong N , et al . Chronic obstructive pulmonary disease in the absence of chronic bronchitis in China. Respirology 2010;15:1072–8. 10.1111/j.1440-1843.2010.01817.x 20723142

[4] de Oca MM , Halbert RJ , Lopez MV , et al . The chronic bronchitis phenotype in subjects with and without COPD: the PLATINO study. Eur Respir J 2012;40:28–36. 10.1183/09031936.00141611 22282547

[5] Jindal SK , Aggarwal AN , Gupta D , et al . Indian study on epidemiology of asthma, respiratory symptoms and chronic bronchitis in adults (INSEARCH). Int J Tuberc Lung Dis 2012;16:1270–7. 10.5588/ijtld.12.0005 22871327

[6] Ferré A , Fuhrman C , Zureik M , et al . Chronic bronchitis in the general population: influence of age, gender and socio-economic conditions. Respir Med 2012;106:467–71. 10.1016/j.rmed.2011.12.002 22197577

[7] Huchon GJ , Vergnenègre A , Neukirch F , et al . Chronic bronchitis among French adults: high prevalence and underdiagnosis. Eur Respir J 2002;20:806–12. 10.1183/09031936.02.00042002 12412668

[8] Peto R , Speizer FE , Cochrane AL , et al . The relevance in adults of air-flow obstruction, but not of mucus hypersecretion, to mortality from chronic lung disease. results from 20 years of prospective observation. Am Rev Respir Dis 1983;128:491–500. 10.1164/arrd.1983.128.3.491 6614643

[9] MRC (Medical Research Council on the Aetiology of Chronic Bronchitis) . Standardized Questionaries on respiratory symptoms. BMJ 1960;2:1665. 10.1136/bmj.2.5213.1665 13688719

[10] NHS inform. Available: www.nhsinform.scot/illnesses-and-conditions/lungs-and-airways/bronchitis

[11] Hodgkin JE , Abbey DE , Euler GL , et al . Copd prevalence in nonsmokers in high and low photochemical air pollution areas. Chest 1984;86:830–8. 10.1378/chest.86.6.830 6499544

[12] Euler GL , Abbey DE , Magie AR , et al . Chronic obstructive pulmonary disease symptom effects of long-term cumulative exposure to ambient levels of total suspended particulates and sulfur dioxide in California Seventh-Day Adventist residents. Arch Environ Health 1987;42:213–22. 3662608

[13] Euler GL , Abbey DE , Hodgkin JE , et al . Chronic obstructive pulmonary disease symptom effects of long-term cumulative exposure to ambient levels of total oxidants and nitrogen dioxide in California Seventh-Day Adventist residents. Arch Environ Health 1988;43:279–85. 10.1080/00039896.1988.10545950 3415354

[14] Holland WW , Reid DD . The urban factor in chronic bronchitis. Lancet 1965;1:445–8. 10.1016/S0140-6736(65)91584-9 14241880

[15] COMEAP . Long-Term exposure to air pollution and chronic bronchitis, 2016. Available: https://www.gov.uk/government/publications/comeap-long-term-exposure-to-air-pollution-and-chronic-bronchitis#:~:text=The%20Committee%20found%20some%20evidence,to%20imply%20a%20causal%20relationship [Accessed 15 Mar 2021].

[16] Heinrich J , Topp R , Gehring U , et al . Traffic at residential address, respiratory health, and atopy in adults: the National German health survey 1998. Environ Res 2005;98:240–9. 10.1016/j.envres.2004.08.004 15820731

[17] Schindler C , Keidel D , Gerbase MW , et al . Improvements in PM10 exposure and reduced rates of respiratory symptoms in a cohort of Swiss adults (SAPALDIA). Am J Respir Crit Care Med 2009;179:579–87. 10.1164/rccm.200803-388OC 19151198

[18] Schikowski T , Ranft U , Sugiri D , et al . Decline in air pollution and change in prevalence in respiratory symptoms and chronic obstructive pulmonary disease in elderly women. Respir Res 2010;11:113. 10.1186/1465-9921-11-113 20727210 PMC2936381

[19] Doiron D , Bourbeau J , de Hoogh K , et al . Ambient air pollution exposure and chronic bronchitis in the lifelines cohort. Thorax 2021;76:772–9. 10.1136/thoraxjnl-2020-216142 PMC8311080 33509968

[20] de Hoogh K , Chen J , Gulliver J , et al . Spatial PM2.5, NO2, O3 and bc models for Western Europe – evaluation of spatiotemporal stability. Environ Int 2018;120:81–92. 10.1016/j.envint.2018.07.036 30075373

[21] de Hoogh K , Gulliver J , Donkelaar Avan , et al . Development of West-European PM_2.5_ and NO_2_ land use regression models incorporating satellite-derived and chemical transport modelling data. Environ Res 2016;151:1–10. 10.1016/j.envres.2016.07.005 27447442

[22] Sunyer J , Jarvis D , Gotschi T , et al . Chronic bronchitis and urban air pollution in an international study. Occup Environ Med 2006;63:836–43. 10.1136/oem.2006.027995 16847030 PMC2078017

[23] Gundersen H , Magerøy N , Moen BE , et al . Low traffic and respiratory symptoms among smoking females: the Hordaland health study. Arch Environ Occup Health 2012;67:189–98. 10.1080/19338244.2011.619214 23074976

[24] Hurley F , Hunt A , Cowie H . Methodology for the cost-benefit analysis for CAFE: volume 2: health impact assessment. AEA Technology Environment 2005.

